# Long-term follow-up study on surgical outcomes of the Faden operation in consecutive esotropia

**DOI:** 10.1038/s41598-020-79582-7

**Published:** 2021-01-11

**Authors:** Suk-Gyu Ha, Seung-Hyun Kim

**Affiliations:** Department of Ophthalmology, Korea University Anam Hospital, Korea University College of Medicine, 73, Inchon-ro, Seongbuk-gu, Seoul, 136-705 Korea

**Keywords:** Diseases, Health care, Medical research

## Abstract

We evaluated the long-term surgical outcomes of medial rectus (MR) recession with the Faden operation in consecutive esotropia (CET). We retrospectively analyzed patients who underwent MR recession with the Faden operation for CET between 2013 and 2018, and compared surgical outcomes between patients who underwent MR recession with the Faden operation (Faden group) and MR recession only (control group). We followed up the patients at 24 months postoperatively. Postoperative success was defined as final deviation of less than distant 5 prism diopters (PD) of eso- or exodeviation at the final visit. We compared postoperative alignment and stereoacuity between the two groups. Stereoacuity was classified as good (60 or better seconds of arc), fair (80–3000 s of arc) or nil. The Faden and control group included 11 and 13 patients, respectively. All patients in the Faden group showed orthophoria and eight patients (72.7%) showed good stereoacuity at the final visit. One patient (9.1%) in the Faden group showed adduction limitation at the final visit. Eight patients (61.5%) in the control group had successful outcomes at the final visit. Six patients (46.2%) in the control group showed good stereoacuity at the final visit. One patient (7.7%) in the control group underwent reoperation for recurrent esotropia at 18 months postoperatively. The surgical outcomes after MR recession with the Faden operation for CET were excellent in long-term follow- up. The Faden operation could be a good surgical option for CET.

## Introduction

Consecutive esotropia (CET) occurs following surgical correction of intermittent exotropia. The incidence of CET after surgery for intermittent exotropia ranges from 6 to 20%^1–3^.

CET generally resolves spontaneously over time, however, persistent esotropia poses a risk of amblyopia, loss of stereopsis in young children, and diplopia in adults^4^. Surgical management should be considered in patients experiencing CET with persistent diplopia^3^.

CET can be augmented by more innervational input to the medial rectus (MR) muscle after bilateral lateral rectus recession^5^. The predisposing factors in the development of CET include strong and variable tonic convergence or tight MR muscle^5,6^. The Faden operation can be used to weaken the rotational force of the rectus muscle when the eye rotates toward the muscle^7^. Excellent short-term results (at postoperative 6 months) of MR recession with the Faden operation have been reported^8^.

The present study evaluated the surgical outcomes of MR recession with the Faden operation in long-term follow-up of more than 2 years.

## Methods

This study was approved by the Institutional Review Board of Korea University Medical Center. It adhered to tenets of the Declaration of Helsinki. Written informed consent was obtained from all patients and their guardians. We retrospectively analyzed the medical charts of patients who underwent MR recession with the Faden operation for CET between January 2013 and March 2018. Patients who had anterior/posterior segment pathology, concomitant cyclovertical muscle surgery, a follow-up duration of less than 24 months after CET surgery, or were older than 15 years at the time of CET surgery were excluded from this study. The ophthalmologic assessments included measurement of the angle of deviation (prism diopters, PD) by alternate cover testing at near (1/3 m) and distant (6 m), ocular movements, such as version and duction, dissociated vertical deviation (DVD) and inferior oblique overaction (IOOA). We evaluated refractive errors by cycloplegic refraction. Refractive errors were calculated as spherical equivalent.

Surgery was performed when esodeviation increased over 15 PD despite nonsurgical management including part-time occlusion and base-out prism treatment. All patients underwent uni- or bilateral MR recession which was combined with the Faden operation since 2016 under general anesthesia by one surgeon (S. H. K). Patients with CET who underwent MR recession with the Faden operation during 2016–2018 were defined as the Faden group, whereas those who underwent MR recession only during 2013–2015 were defined as the control group. The surgical amount of MR recession was decided based on the preoperative angle of deviation. Bilateral MR recession was performed for esodeviation of > 30 PD (Table 1). For unilateral surgery, we combined the Faden operation with MR recession. We performed the Faden operation in the deviating eye only for cases undergoing bilateral MR recession. During MR recession with the Faden operation, the MR was removed from the original insertion, and a posterior fixation suture (Faden operation) was placed through the sclera 10–12 mm posterior to the MR insertion in the center of the arc of contact using a non-absorbable suture.

**Table 1:** Surgical dosages of medial rectus muscle recession.

Prism diopters	Unilateral recession (mm)	Bilateral recession (mm)
15	5	
20	6	
25	6.5	
30		4.0/5.0
35		5.0/5.0
40		5.0/6.0
45		6.0/6.0
50		6.5/6.5

**Figure 1:**
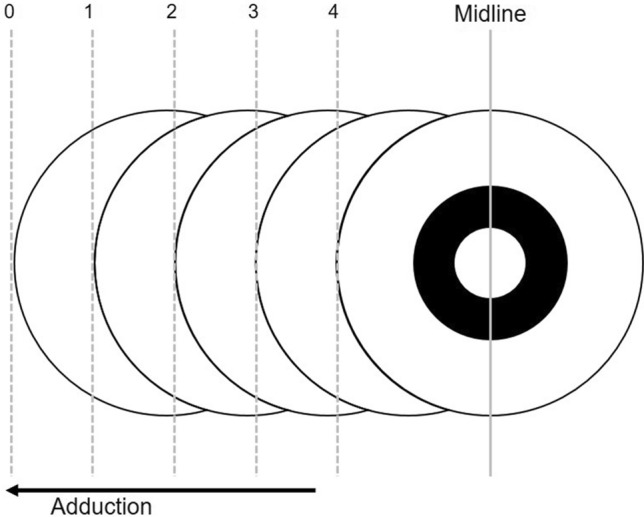
Schematic diagram for grading abduction limitation. A grade of 0 was noted for full excursion, 4 for 0% excursion just reaching midline, and 3 to 1 for 25% increments.

All patients were followed up postoperatively at 1 day, 1 week, one month, 3 months, and 6 months, and at 6-month intervals until the final visit. We included the patients who were followed up at 24 months after surgery. At each visit, we evaluated the angle of deviation (PD). We also assessed near stereoacuity using the Titmus Test (Stereo Optical Co., Inc., Chicago, IL, USA) at 1/3 m. The Near stereoacuity was classified into three groups as follows: good (60 or better seconds of arc), fair (80–3000 s of arc,) or nil. Distant fusion was assessed with a Vectogram (Luneau L29 chart projector; Luneau SAS, Chartres, France) at 6 months. The limitation of adduction in the Faden group was evaluated at postoperative follow-up visits. Adduction limitation was quantitatively scored for adduction by the severity of limitation on a grading scale of 0 to 4. A grade of 0 was noted for full excursion, 4 for 0% excursion just reaching midline, and 3 to 1 for 25% increments (Fig. 1).

We compared baseline ophthalmologic findings between the Faden and the control group. We evaluated postoperative alignment (PD) and sensory outcomes at 24 months postoperatively. Postoperative success was defined as a final deviation of less than distant 5 PD of eso- or exodeviation at the final visit.

Statistical analyses were performed using SPSS for Windows (V.18.0; SPSS, Inc, Chicago, IL, USA). We used Mann–Whitney U tests to compare continuous variables and evaluated the associations between categorical variables using Fisher’s exact test. A *p* < 0.05 was considered statistically significant.

## Results

This study included 24 patients. The Faden and control group included 11 and 13 patients, respectively. The mean patient age at operation was 5.7 ± 2.5 years (range 4–9 years), and 19 patients (41.7%) were male patients. The preoperative distant and near angles of esotropia were 25.0 ± 11.9 PD (range 15–55 PD) and 25.8 ± 12.1 PD (range 15–55 PD), respectively. Seven patients (36.8%) underwent bilateral MR recession for CET. The Basic demographics of the subgroups are described in Table 2.

**Table 2:** Basic patient demographics.

	Faden group* (n = 11)	Control group** (n = 13)	*p*
Age at operation for CET (years)	7.3 ± 1.4 (5–9)	5.5 ± 1.6 (4–8)	0.11^†^
Male (%)	5 (45.5)	5 (38.5)	0.56^‡^
Visual acuity (logMAR)	0.04 ± 0.08 (0–0.2)	0.03 ± 0.07 (0–0.2)	0.21^†^
Refractive error***	− 2.7 ± 1.4 (− 5.0 to 2)	− 3.0 ± 0.8 (− 4.5 to 1)	0.09^†^
**Prior surgery (%)**			0.16^‡^
Unilateral LR recession	1 (9.1)	2 (15.4)	
Bilateral LR recession	10 (90.1)	11 (84.6)	
IOOA, DVD	–	–	–
Duration of CET (months)	7.0 ± 4.3 (2–18)	8.7 ± 9.3 (3–28)	0.15^†^
**Preoperative esodeviation, PD**			
Distant	26.3 ± 13.1 (15–55)	23.0 ± 9.2 (15–40)	0.21^†^
Near	26.3 ± 13.1 (15–55)	23.9 ± 10.1 (15–40)	0.22^†^
BMR recession (%)	3 (23.1)	4 (30.8)	0.14^‡^
Amount of MR recession (mm)	5.9 ± 4.3 (5–6.5)	5.6 ± 5.1 (5–6)	0.34^†^

*Medial rectus recession with the Faden operation, **Medial rectus recession, ***calculated as spherical equivalent, minus value means myopia. ^†^Mann–Whitney U test, ^‡^Fisher exact test.

*CET* consecutive esotropia, *LR* lateral rectus ^‡^prism diopters, *IOOA* inferior oblique overaction, *DVD* dissociated vertical deviation, *BMR* Bilateral medial rectus, *MR* medial rectus.

At postoperative 24 months, all patients in the Faden group had achieved orthophoria at distantance and near. In the control group, two patients (15.4%) had esodeviation > 5 PD and three patients (23.1%) had exodeviation > 5 PD. One patient (7.7%) in the control group required part-time patching for esodeviation. One patient (7.7%) in the control group underwent reoperation for recurrent esotropia at 18 months postoperatively. There was a significant difference the angle of deviation between the two groups at 24 months postoperatively (*p* = 0.04, Table 3). In the Faden group, eight patients (72.7%) showed good stereoacuity and three patients (27.3%) showed fair stereoacuity at 24 months postoperatively. In the control group, six (46.2%), five (38.5%), and two (15.4%) patients showed good, fair, and nil stereoacuity, respectively (*p* = 0.02, Table 3).

**Table 3:** Surgical outcome at postoperative 24 months in the two groups.

	Faden group* (n = 11)	Control group** (n = 13)	*p*
**Angle of deviation at distant, PD**^**†**^		6.5 ± 10.4 (0–35)	< 0.01^†^
Orthotropia (%)	11 (100)	5 (38.5)	0.04^‡^
> 5 PD of exodeviation (%)		2 (15.4)	
> 5 PD of esodeviation (%)		2 (15.4)	
**Stereoacuity, sec of arc**			
Good (60 or better) (%)	8 (72.7)	6 (46.1)	0.02^‡^
Fair (80–3000) (%)	3 (27.3)	5 (38.5)	
Nil (%)		2 (15.4)	
Reoperation (%)	–	1 (7.6)	

*Medial rectus recession with The Faden operation, **Medial rectus recession, ^†^Mann–Whitney U test, ^‡^Fisher exact test.

*PD* prism diopter.

**Figure 2:**
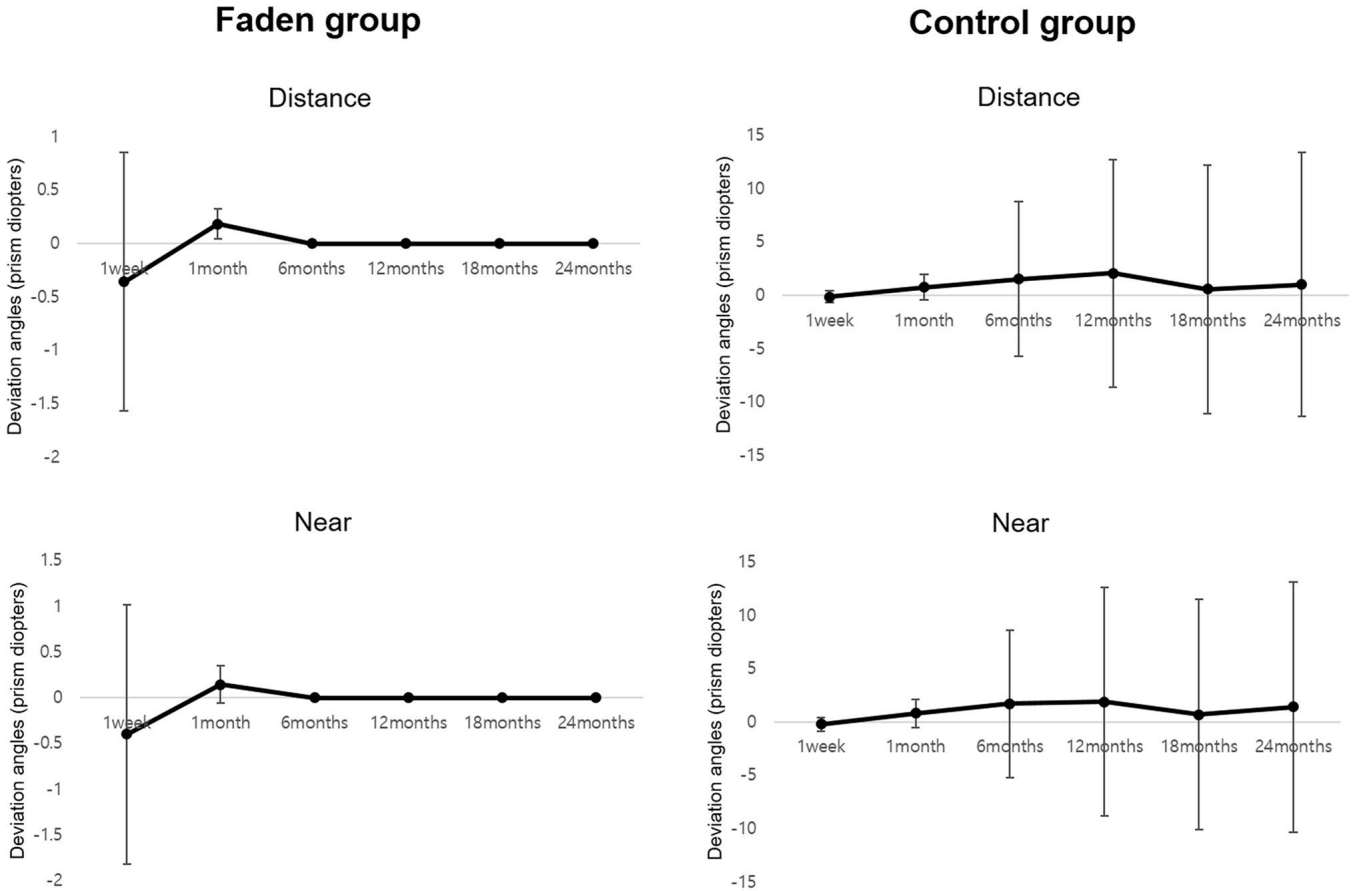
Angle of deviation during postoperative follow-up. Faden group: medial rectus recession with the Faden operation, Control group: medial rectus recession.

In the Faden group, all patients showed orthophoria without any deviation in the final visit. During the postoperative follow-up period, there was no small angle of deviation greater than 5 PD. However, there was a greater mean standard deviation of the angle of deviation in the control group throughout the follow-up period (Fig. 2). In the Faden group, we observed adduction limitations greater than grade 0 in two patients (18.1%) at postoperative 18 months (grade = 1, 2 patients); however, there was only one patient (9.1%) with adduction limitation (grade = 1) without subjective diplopia at the final visit.

**Figure 3:**
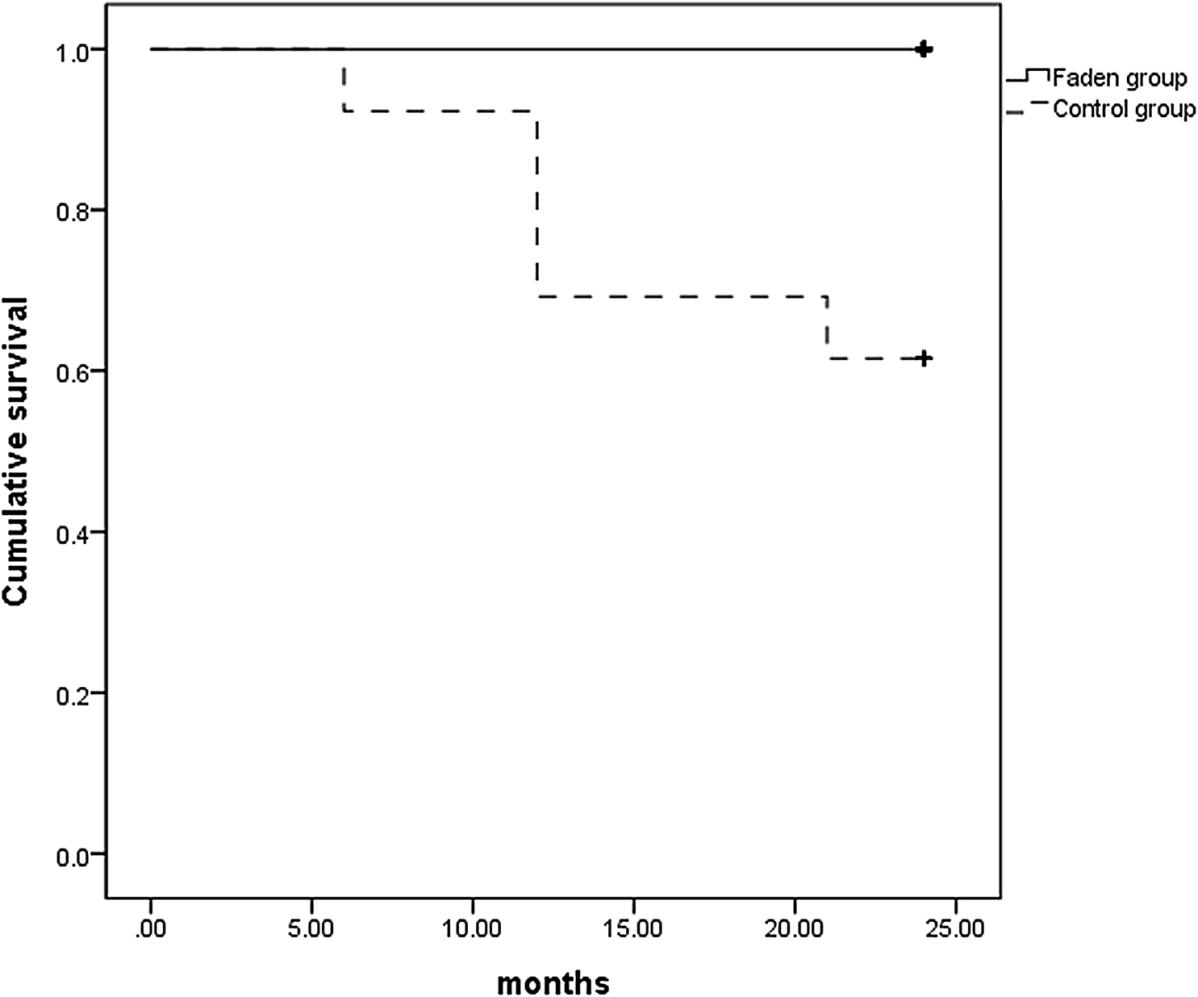
Kaplan Meier survival analysis showing the cumulative probability of success rate in the two groups at 24 months postoperatively.

The Kaplan Meier survival curves for surgical success showed successful outcomes for all patients in the Faden group, and for eight patients (61.5%) in the control group at 24 months postoperatively (*p* < 0.01, Fig. 3).

## Discussion

In this study as well as in a previous study^8^, MR recession with the Faden operation showed excellent surgical results in all patients with CET. In 24 months of follow-up after surgery, the patients who underwent MR recession with the Faden operation maintained stable alignment and good stereoacuity.

There remains no clear reason for the occurrence of CET after surgery for exotropia. However, in some patients, this initial overcorrection may persist and treatment may be required because of diplopia in adults and some children and the risk of amblyopia and loss of binocularity in children^4^. Patients with large overcorrection resultings in CET require further surgery or nonsurgical treatment, such as prisms or Botox injection^9^.

The optimal surgical treatment for CET has not yet been firmly established. Park et al.^10^ reported the surgical outcomes of unilateral lateral rectus advancement with medial rectus recession (A&R group) and bilateral medial rectus recession (BMR group) for CET. At 12 months postoperatively, successful surgical outcomes were noted in 12 patients (85.7%) from the A&R group and 11 patients (73.3%) from the BMR group^10^. In the present study, all cases who underwent MR recession with the Faden operation achieved orthophoria without standard deviation at postoperative 24 months.

There is no consensus regarding the pathogenic convergent mechanism of CET. The tonic part of convergence changes with time^11^ and is responsible for the variance in esodeviation. We believe that the effect of the recession was augmented in this study, since the Faden operation may add a globe stabilization effect without significantly increasing the limitation of movement. Therefore, MR recession combined with the Faden operation may be appropriate for patients with CET who have strong and variable tonic convergence.

Kim et al.^8^ reported the surgical results of MR recession with the Faden operation for CET at 6 months postoperatively, which the patients maintained orthophoria and good stereoacuity (60 or better seconds of arc) at the final visit. In the present study, all patients in the Faden group had maintained orthophoria and good stereoacuity (60 or better seconds of arc) at postoperative 24 months. Only one patient showed a small degree of adduction limitation after the Faden operation at 24 months postoperatively. We believe that a small degree of adduction limitation such as diplopia in lateral gaze in daily life is not a problem, the patient’s original deviation was exotropia.

This study has some limitations. First, it was a retrospective design and included a small number of cases. However, CET after surgery is generally not common, and surgical management is considered in patients with CET after at least several months have elapsed. Thus, a relatively small number of patients could be enrolled in this study. Second, we included patients with bilateral or unilateral MR recession. Other surgical methods, such as lateral rectus advancement or combined resection/resection surgery, were not compared.

In conclusion, excellent surgical outcomes of MR recession with the Faden operation were observed and maintained in all patients with consecutive ET in the long-term follow-up.
